# Ultra-small bimetallic iron–palladium (FePd) nanoparticle loaded macrophages for targeted tumor photothermal therapy in NIR-II biowindows and magnetic resonance imaging†

**DOI:** 10.1039/c9ra05649a

**Published:** 2019-10-17

**Authors:** Yang Yang, Mng Lyu, Jing-Hua Li, Dao-Ming Zhu, Yu-Feng Yuan, Wei Liu

**Affiliations:** Key Laboratory of Artificial Micro- and Nano-Structures of Ministry of Education, School of Physics and Technology, Wuhan University Wuhan Hubei 430072 China wliu@whu.edu.cn; Department of Hepatobiliary and Pancreatic Surgery, Zhongnan Hospital of Wuhan University Wuhan Hubei 430071 China yuanyf1971@whu.edu.cn; Medical Science Research Center, Zhongnan Hospital of Wuhan University Wuhan Hubei 430071 China

## Abstract

Nanoparticles working in the NIR-II biowindows possess larger maximum permissible exposure (MPE) and desirable penetration depth to the laser. However, most NIR-II responsive nanomaterials lack tumor targeting and Magnetic Resonance Imaging (MRI) ability. This greatly limits their applications. This study reported ultra-small bimetallic iron–palladium (FePd) nanoparticle loaded macrophages for targeted tumor photothermal therapy in NIR-II biowindows and magnetic resonance imaging. The crystal phase, morphology, absorption spectrum and photothermal performance of the synthesized samples were systematically characterized. The effects of photothermal therapy and nuclear magnetic imaging (MRI) were studied both *in vitro* and *in vivo*. Since FePd nanoparticles have both iron and palladium elements, it had a good MRI imaging capability and high photothermal conversion efficiency (36.7%). After binding to macrophages, FePd nanoparticles@macrophages (FePd@M) showed a good tumor targeting ability and were used for targeting NIR-II photothermal therapy and MRI imaging of tumors. The results of photothermal treatment showed that the tumor volume decreased by 90% compared to the control group, and no significant organ toxicity was observed. The results of MRI imaging showed that the FePd@M has the best imaging effect. The nanoparticles with the excellent NIR-II PTT ability and MRI effect have overcome the problem of tumor targeting and avoid the rapid removal of ultra-small nanoparticles. The FePd@M delivery system provides new ideas for material construction in the NIR-II region and has great clinical application potential.

## Abstract 2

Nanoparticles working in the NIR-II biowindows possess larger maximum permissible exposure (MPE) and desirable penetration depth to the laser.

## Graphical abstract

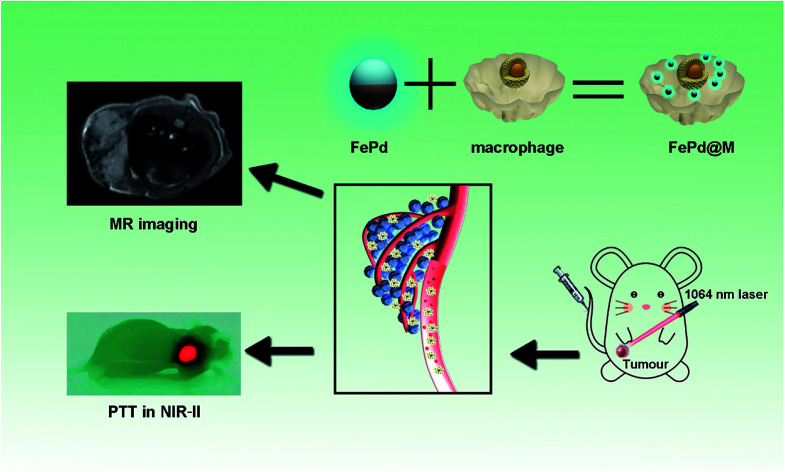

## 1. Introduction

Cancer is still a serious public health problem in the world.^1^ The top three cancers by mortality rate are lung cancer, liver cancer, and gastric cancer.^2^ Among them, hepatocellular carcinoma (HCC) is a terrible malignant tumor, and 500 000 people die of liver cancer every year in the world.^3^ However, conventional chemotherapy, as the most common treatment, often causes patients to suffer due to systemic toxicity and side effects.^4^ There is an urgent need for new and more effective treatment strategies for HCC.

The development of some nanomaterials with unique physical and chemical properties provides an opportunity to design multifunctional nanoscale platforms.^5^ At present, the nanotechnology treatment has brought unprecedented opportunities for the precise treatment of cancer, with small side effects and good therapeutic effect.^6^ In particular, nanomaterial-mediated photothermal therapy (PTT) can locally transform the near-infrared (NIR) light transmitted by the tissue into thermal ablation of cancer cells.^7,8^ PTT is less invasive and requires lower intensity radiation, which is more selective for cancer cells.^9^ The near-infrared photothermal therapy is a very promising treatment with the advantages of high efficiency, minimal invasiveness, and strong selectivity.^10–12^ Especially in the near-infrared two-region biological window (NIR-II, 1000–1350 nm), the blood and soft tissue absorption are the smallest, and the imaging resolution and light penetration depth are greatly improved.^13^ More and more attention is devoted to this area enables people to explore many nanomaterials respond to the NIR-II window, including transition metals sulfide/oxide semiconductor,^14–16^ precious metal nanomaterials,^17–19^ and polymer nanocomposites.^20^ At the same time, these nanomaterials usually have photoacoustic imaging and computerized tomography (CT) imaging capabilities.^18,21,22^ Tumor-targeted precision imaging is also the key to accurate tumor therapy.^23^ However, most NIR-II responsive nanoparticles lack tumor targeting and magnetic resonance imaging ability, which greatly limits their application.

**Fig. 1:**
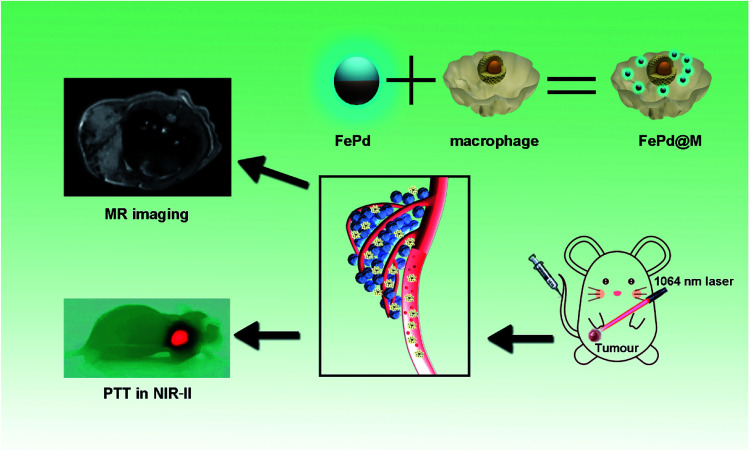
Ultra-small iron–palladium (FePd) nanoparticles with Near-Infrared-II (NIR-II) region photothermal responsive for targeted tumor photothermal therapy and magnetic resonance imaging.

Here, we have designed an ultra-small bimetallic iron–palladium (FePd) nanoparticles loaded macrophages for targeted tumor photothermal therapy in NIR-II biowindows and magnetic resonance imaging as shown in Fig. 1. This nanoparticles with a particle size of about 4 nm have both strong NIR-II photothermal absorption capacity (about 45% photothermal conversion efficiency) and good Magnetic Resonance Imaging (MRI) capabilities. What's more, the ultra-small size (<6 nm) of nanoparticles let it be eliminated renally.^24^ Therefore, FePd NPs are a biocompatible nanomaterial. However, nanoparticles such a small size range (<20 nm) are rapidly aggregated in blood, passivated by serum protein adsorption onto the nanoparticle surfaces, losing therapeutic potentials.^25–28^ In order to improve the targeting of the FePd NPs to tumor tissues, macrophages are used as carriers to deliver FePd NPs in our study. Macrophages have been proven as an excellent nanoparticles carrier with small side effects and good tumor targeting.^29–32^ FePd NPs loaded macrophages (FePd@M) can be targeted to the tumor site, which also greatly increased the accumulation of nanoparticles in the tumor site. Compared with the control group and exposed nanoparticles, FePd@M showed a stronger photothermal treatment effect (about 90% of tumors are ablated) and better nuclear magnetic imaging. Our work provides new ideas for designing NIR-II response nanoparticles, and the multifunctional FePd@M compound has good clinical application potential.

## 2. Results and discussions

### Preparation and characterization of FePd nanoparticles

**Fig. 2:**
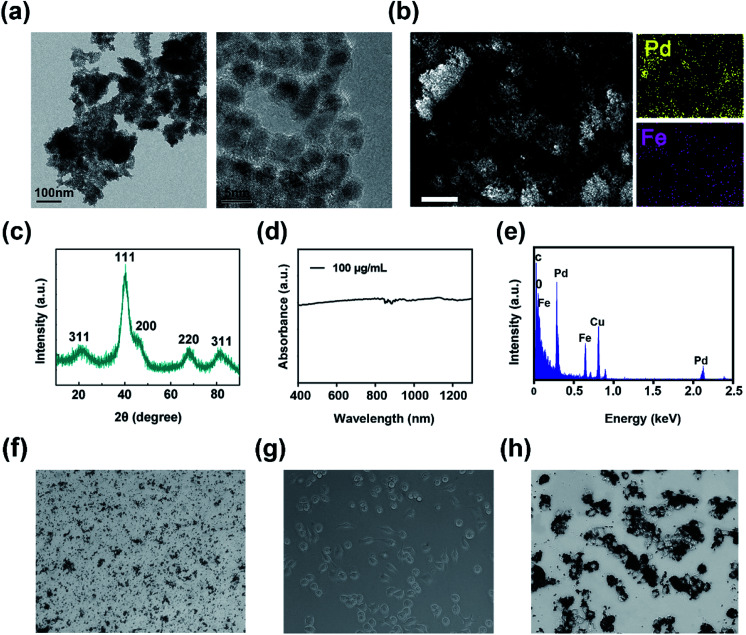
Characterization of FePd nanoparticle: (a) TEM image of FePd NPs with different magnifications. (b) SEM image and corresponding elemental mapping images of Fe and Pd. Scale bars = 1 μm. (c) XRD spectra; (d) absorbance spectra and (e) EDS spectra of FePd NPs. (f) Bright-field image of pure FePd NPs. (g) Bright-field image of macrophages. (h) Bright-field image of FePd@M.

Transmission electron microscopy (TEM) was employed to visually the structure of FePd nanoparticles, as shown in Fig. 2a and S1,† it revealed that the FePd nanoparticle have a uniform size distribution and the size of the FePd nanoparticle is about 3.8 nm. The XRD patterns as shown in Fig. 2c, it demonstrated peaks of {111}, {200}, {220} and {311} facet. The obvious lattice fringe in HRTEM image as shown in Fig. S2† and revealed an interplanar spacing of 0.230 nm in correspondence to the {111} lattice plane of Pd phase. The optical absorption spectra of the FePd nanoparticle and FePd@M are acquired on a UV-visible-NIR spectrophotometer. FePd NPs have good absorption in the NIR-II region (Fig. 2d), and FePd@M also has good stability (Fig. S3†). As shown in Fig. 2b, the Fe and Pd element mapping pattern from FePd nanoparticles further proved that it was successfully prepared. EDS spectra of FePd NPs showed an atomic content is 42.8% for Fe and 57.2% for Pd (Fig. 2e). Bright-field images of pure nanoparticles and macrophages before and after uptake of FePd nanoparticles as shown in Fig. 2f–h.

### *In vitro* photothermal ability of FePd nanoparticles

**Fig. 3:**
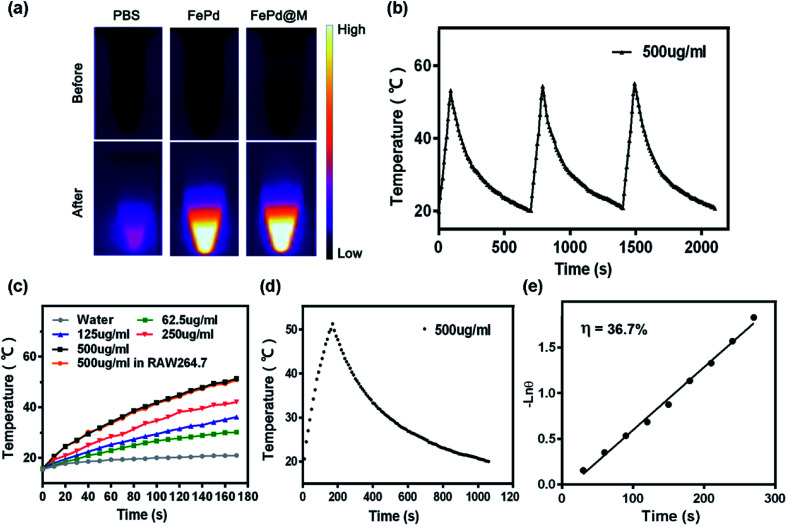
*In vitro* photothermal ability of FePd nanoparticles. (a) The infrared thermal images of pure water and FePd nanoparticles (500 μg ml^−1^) irradiated for 0 and 3 min (1064 nm, 1.0 W cm^−2^). (b) The photothermal response of FePd nanoparticles aqueous solution (500 μg ml^−1^) with a NIR laser (1064 nm, 1.0 W cm^−2^), and then the laser was shut off. Repeat three times. (c) Temperature elevation of water and FePd nanoparticles aqueous solutions with different concentrations as a function of irradiation time exposure to 1064 nm NIR laser (1.0 W cm^−2^). The temperatures were measured. Every 10 s using a thermocouple microprobe. (d) The photothermal response of FePd nanoparticles aqueous solution (500 μg ml^−1^) with a NIR laser (1064 nm, 1.0 W cm^−2^), and then the laser was shut off. (e) Linear time data *versus* −ln *θ* obtained from the cooling period of (d).

We verified the *in vitro* photothermal conversion capability of FePd nanoparticles and FePd@M. As shown in Fig. 3a and c, the nanoparticles showed good heating capacity under 1064 nm laser irradiation and could rise up to more than 50 °C in 5 minutes. The greater the concentration, the more obvious the effect of temperature rise. It is also worth noting that the temperature risen of macrophages carrying the same quality materials is consistent with that of pure materials, indicating that the photothermal conversion ability of nanoparticles has not changed after macrophage phagocytosis. Subsequently, we verified its photothermal cycling ability (as shown in Fig. 3b). The results show that the FePd nanoparticles have good photothermal cycling ability and can be repeatedly heated by the laser. We further calculated the photothermal conversion efficiency of the material, and the photothermal conversion efficiency of the FePd nanoparticles was about 36.7% (Fig. 3d and e), this was calculated according to the method established by Roper and co-workers^33^ and indicated good photothermal properties of FePd NPs. In general, FePd nanoparticles have good photothermal conversion capability in the NIR-II optical window and can be used for photothermal therapy.

### The biocompatibility and cellular uptake of FePd nanoparticles

**Fig. 4:**
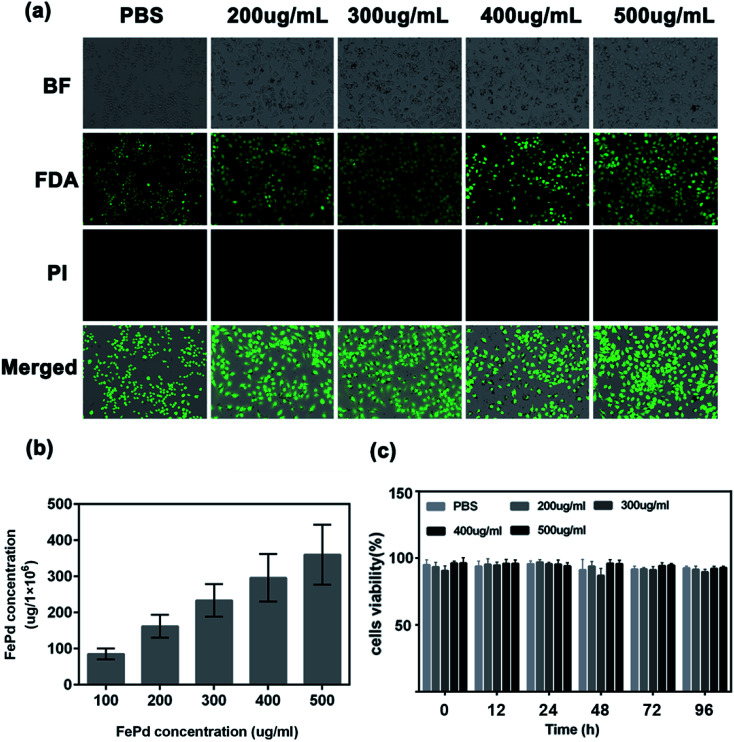
The biocompatibility of the FePd nanoparticles: (a) FDA/PI staining (viable cells are stained green with FDA and dead cells are stained red with PI) after the macrophages were incubated with PBS and FePd nanoparticles of different concentration (200, 300, 400 and 500 μg ml^−1^) for 24 hours; (b) quantitative determination of macrophages uptake of FePd; (c) cell viability of RAW 264.7 cells determined by CCK-8 assay after incubating with different concentrations of FePd NPs for 0, 24, 36, 48, 72 and 96 h.

Good biocompatibility of nanomaterials is an essential prerequisite for biomedical applications.^34^ To evaluate the biocompatibility of FePd nanoparticles, FDA/PI live/dead cell staining was used, live cells were stained green by FDA, and dead cells were stained red by PI. From the fluorescence diagram in Fig. 4a and S4,† after the macrophages were incubated with PBS and FePd nanoparticles of different concentration (200, 300, 400 and 500 μg ml^−1^) for 24 and 48 hours (Fig. S3†), substantially no red fluorescence showing dead cells appeared. At the same time, flow cytometry (Fig. S5†) and CCK-8 (Fig. 4c) assay was also performed. The apoptotic flow results showed that macrophages did not apoptosis even in the presence of high concentrations of FePd NPs (500 μg ml^−1^), indicating that macrophages have the ability to carry FePd NPs, and FePd nanoparticles didn't cause damage to cells. FePd NPs have a good biocompatibility.

Phagocytosis of FePd nanoparticles by macrophages is an important condition for macrophages as a good carrier.^30^ In order to quantitatively determine the phagocytosis of FePd NPs by macrophages, we used ICP-AES to detect the content of iron and palladium in cells. As shown in Fig. 4b, macrophages were able to efficiently phagocytose FePd nanoparticles after being cultured with different concentrations of FePd nanoparticles. The experimental data show that when the concentration of FePd nanoparticles is 500 μg ml^−1^, the phagocytosis of the cells is about 360 μg, which shows the high phagocytic efficiency of macrophages to FePd nanoparticles.

### *In vivo* biodistribution and tumor targeting of FePd@M

**Fig. 5:**
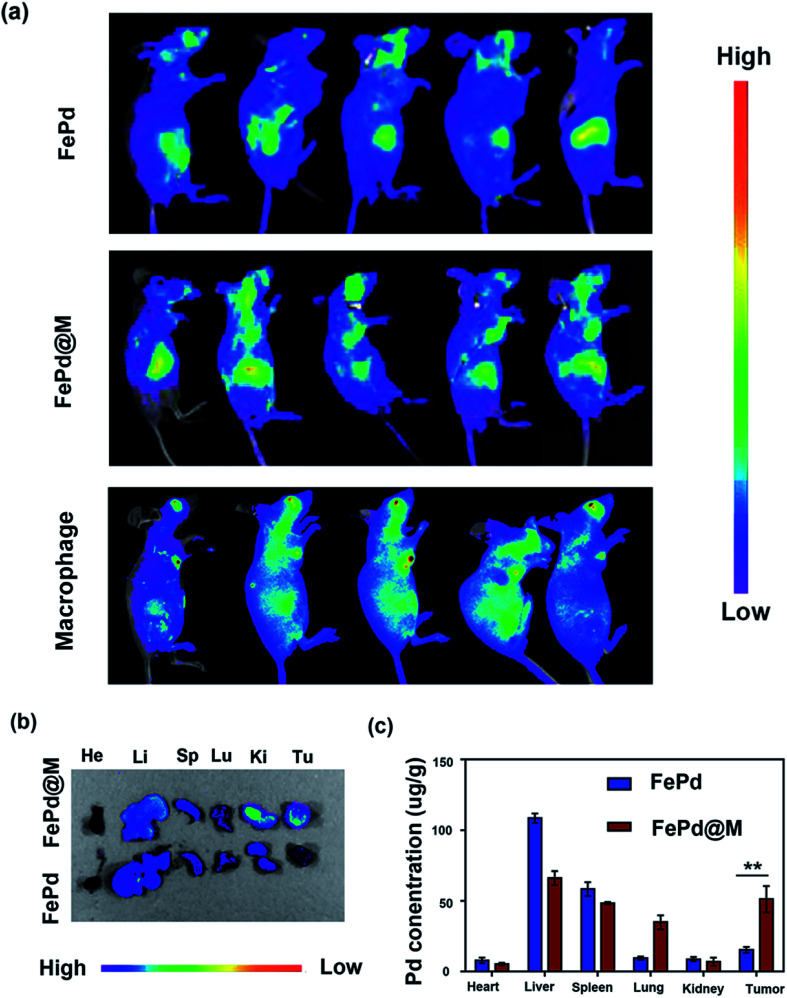
*In vivo* biodistribution of FePd and FePd@M in mice bearing Huh-7 tumors: (a) *in vivo* fluorescence tracking of the Cy5-labeled FePd, pure macrophage and FePd@M after intravenous injection; (b) *ex vivo* fluorescence images of the tumors and major organs including heart (He), liver (Li), spleen (Sp), lung (Lu), and kidney (Ki) at 24 h after intravenous injection of dye-labeled FePd and dye-labeled FePd@M; (c) biodistribution of Pd at 24 h post-injection of the FePd and FePd@M.

The most critical factor for the use of nanoparticles for tumor treatment or imaging is the amount of accumulation at the tumor site. The body's immune system removes most of the nanoparticles, so it is urgent to develop nanoparticles that target tumor sites. Therefore, we have studied the distribution *in vivo* and tumor targeting of FePd@M nanoparticles. To validate FePd@M *in vivo* distribution and tumor targeting capabilities, *In vivo* imaging system (IVIS) and organ *ex vivo* imaging was used. Firstly, macrophages and FePd NPs were co-cultured to form FePd@M, and then injected into the mice implanted with human liver cancer cell line Huh-7 tumors by tail vein. The pure FePd NPs were injected into the Huh-7 tumor bearing mice as a control group. As shown in the Fig. 5a, pure macrophage and FePd@M both have a large accumulation in the liver of mice, which is due to the reticuloendothelial systems (RES) absorption.^30^ The FePd@M showed a very bright Cy5 fluorescence at the tumor site, indicating that the FePd@M also accumulate in the tumor site, and the pure material accumulates very little in the tumor site. Subsequently, we performed imaging of main organs after the mice were sacrificed as shown in Fig. 5b. It was found that the FePd@M had a stronger accumulation at the tumor site than the pure NPs, corresponding to the results of *in vivo* imaging. To further validate the targeting of FePd@M to tumor tissue, we quantitatively tested the content of nanomaterials in each organ through ICP-AES. As shown in the Fig. 5c, for the pure FePd NPs group, we detected high concentrations of palladium ions in the liver and spleen, and the concentration at the tumor site was low (15.26 ± 2.08 μg g^−1^). This is probably because ultra-small nanoparticles are easily removed *in vivo*, and the EPR effect at the tumor site is relatively weak.^35^ On the contrary, in addition to the higher accumulation in the liver and spleen, FePd@M also have a very strong accumulation in the tumor site (51.10 ± 9.34 μg g^−1^), and the concentration of palladium ions in the tumor site is 3–4 times larger than that of the pure NPs group. In general, FePd@M have a good tumor targeting ability and avoid the fatal defects of ultra-small nanoparticles.

### *In vitro* and *in vivo* MRI

**Fig. 6:**
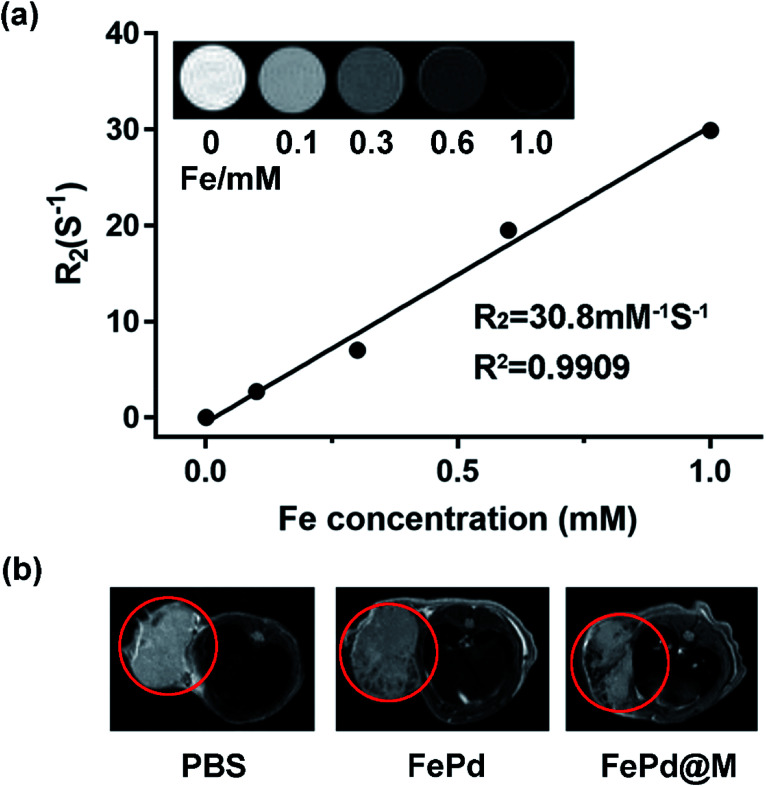
*In vitro* and *in vivo* MRI imaging. (a) *T*_2_ relaxation rate values (*R*_2_) of FePd nanoparticles at different concentrations. The inset shows *R*_2_ and *R*_2_ value of FePd nanoparticles and its T_2_-weighted MR images at different Fe concentrations. (b) Representative *in vivo T*_2_-weighted MR images of Huh-7 tumor-bearing mice before and after injection of PBS, FePd, FePd@M.

The nanoparticles currently available for nuclear magnetic imaging include Fe_3_O_4_, MnO_2_, Gd^3+^-containing polymers, *etc.* However, due to the tumor targeting and single function, the application is greatly limited. Therefore, it is urgent to synthesize a multifunctional nanoparticle to target tumor magnetic imaging. Our synthetic FePd nanoparticles which contain iron, may be used as a novel nuclear magnetic imaging contrast agent, combined with the targeting ability of macrophages, we have verified the MRI *in vivo* and *in vivo* capabilities of FePd@M. Firstly, we use the MRI system to measure the *R*_2_ value of FePd nanoparticles. As shown in the Fig. 6a, as the concentration of FePd nanoparticles increased, the *T*_2_ image gradually becomes black, the relaxation time is shortened, and the relaxation rate is increased. The value of the *T*_2_-weighted relaxation rate *R*_2_ of FePd nanoparticles is 30.80 mM^−1^ S^−1^. It shows that the material has better MRI capability and is concentration dependent and can be further used for nuclear magnetic imaging *in vivo*.

To evaluate nuclear magnetic imaging *in vivo*, when the tumor volume carrying Huh-7 mice grew to approximately 400 mm^3^, we injected 200 μl of FePd (1 mg ml^−1^) and FePd@M (containing 1 mg ml^−1^ FePd) by tail vein injection. Then we used the MRI system to detect the MRI of the tumor site. As shown in Fig. 6b, the MRI image showed that the MR image of the tumor site of the pure FePd was darker than that of the PBS group, and the MRI signal in the FePd@M group tumor site was stronger than the PBS and FePd groups. It indicates that the FePd@M group has a good enrichment ability at the tumor site and can be used for nuclear magnetic imaging targeting tumor tissues.

### *In vivo* photothermal therapy

**Fig. 7:**
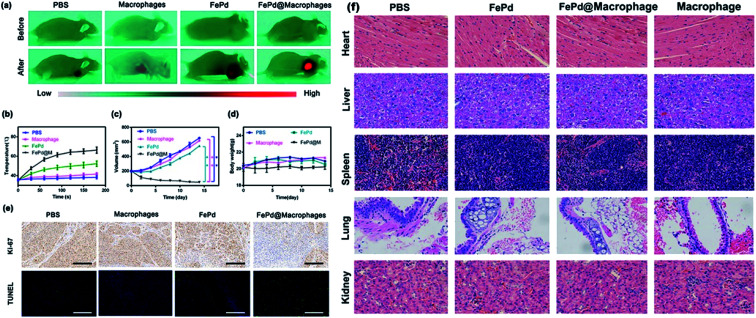
*In vivo* photothermal therapy and cytotoxicity. (a) Thermal images of Huh-7 tumor-bearing mice after injection of PBS, FePd, macrophages, FePd@macrophages and exposure to 1064 nm laser irradiation. (b) Heating curves of Huh-7 tumors upon 1064 nm laser irradiation after injection of PBS, FePd, macrophages, FePd@macrophages as a function of irradiation time. (c) Tumor volume curves after injection of PBS, FePd, macrophages, FePd@macrophages and exposure to 1064 nm laser irradiation. (d) Treatment side effects were assessed by mice body weight in PBS, FePd, macrophages, FePd@macrophages group. (e) Representative TUNEL and Ki-67 stained tumor slice images of mice after injection of PBS, FePd, macrophages, FePd@macrophages and exposure to 1064 nm laser irradiation. Scale bars = 100 μm. (f) Histopathologic examination of the tissues including heart, liver, spleen, lung and kidney from BALB/c nude mice after intravenous administration of PBS, FePd (500 μg ml^−1^) and FePd@M (containing 500 μg ml^−1^ FePd) with NIR irradiation for 20 d. Scale bars = 100 μm.

Currently photothermal therapy is a widely studied therapy. The reagents commonly used in photothermal therapy include precious metal nanoparticles,^36–40^ small molecule reagents,^41–43^ carbon based nanomaterials,^44–46^*in vivo* photothermal effect is the most direct verification method for evaluating nanoparticles for photothermal therapy, so we verified the photothermal treatment of FePd@M nanoparticles *in vivo*. We randomly divided BALB/c nude mice bearing Huh-7 liver tumor into four groups. When the tumor volume of BALB/c nude mice grew to about 200 mm^3^, each mouse was injected with 200 μl of PBS solution, pure macrophages, pure FePd nanoparticles (500 μg ml^−1^) and FePd@M (containing 500 μg ml^−1^ FePd) through the tail vein. After 24 hours, the mice were anesthetized and irradiated with a 1064 nm laser (1.0 W cm^−2^) for 3 min at the tumor site for photothermal therapy. At the same time, an infrared camera is used to monitor the temperature change of the tumor site. As shown in Fig. 7a and b, there was no significant change in the temperature of the tumor site in the PBS group and macrophages group, and the temperature of the tumor site in the FePd group increased by about 10 °C, while the temperature of the tumor site in the FePd@M group increased significantly, and increased to about 60 °C after 3 minutes. It is indicated that the nanoparticles have good photothermal conversion efficiency and can be heated to a temperature exceeding the tolerance of the cells (42 °C).^22,47^ In addition, the FePd@M group had a better temperature-increasing effect than the FePd group, indicating that the FePd@M was abundantly enriched in the tumor site of the mouse, and the photothermal effect was more obvious.

In order to evaluate the anti-tumor effect of FePd@M after photothermal treatment of tumor sites in mice, we measured the body weight and tumor volume of each group every two days. As shown in Fig. 7c, rapid tumor growth was obtained in the PBS group, macrophages group, and the tumor grew from about 200 mm^3^ to 650 mm^3^. While the tumor volume was inhibited by 15% in the FePd group, tumor volume was inhibited by 90% in the FePd@M group. Due to its good targeting, FePd@M can be largely accumulated in the tumor site. Under laser irradiation, the caused high temperature is enough to kill a large number of cancer cells. However, the pure FePd nanoparticles can't be accumulated in the tumor site effectively, so the treatment effect is not obvious. To further verify the destruction of tumor tissue, we stained TUNEL and Ki67 for tumor tissue of each group. As shown in Fig. 7e, the FePd@M group had the greatest degree of apoptosis and minimal cell proliferation compared to the other groups, it is further explained that FePd@M has good photothermal therapeutic effect. It should be noted that there was no significant change in body weight in all treatment groups and indicating good biocompatibility of FePd@M (Fig. 7d).

Similarly, 4T1 tumor-bearing BALB/c mice were used to evaluate the photothermal effect of FePd in other models. As shown in Fig. S6,† we verified the photothermal treatment of FePd@M nanoparticles *in vivo* of 4T1 tumor-bearing BALB/c mice. As shown in Fig. S6(a),† in comparison, the tumor temperature under 1064 nm NIR laser irradiation showed the FePd@M group has a very slight change, and increased to about 65 °C. The tumor volumes of the four groups were measured every 2 days using a digital caliper, as shown in Fig. S6(b),† in the groups of PBS, macrophage, FePd group, the volumes reaching 700 mm^3^, while the FePd@M group was completely eradicated. As shown in Fig. S6(c),† no significant difference in body weight was observed between the treatment group and the control group within 14 days, indicating that the cells and nanoparticles had no toxic and side effects on the mice. Tumor slice images as shown in Fig. S6(d),† the FePd@M group had the greatest degree of apoptosis, further proof, FePd@M group has a good photothermal treatment effect.

### *In vivo* cytotoxicity evaluation

In order to evaluate the potential toxicity of FePd nanoparticles, histopathological analysis methods were used to detect the toxicity *in vivo*. After 15 days, the mice were sacrificed and the main organs such as heart, liver, spleen, lung and kidney were taken for H&E staining. As shown in Fig. 7f, it is apparent that no significant organ damage or inflammatory damage was observed in the control group and the experimental group throughout the treatment period, indicating that the FePd nanoparticles have good biocompatibility *in vivo*.

## 3. Conclusions

Overall, we have successfully designed an ultra-small multifunctional FePd nanoparticles with Near-Infrared-II (NIR-II) photothermal response for targeted tumor photothermal therapy and magnetic resonance imaging. A series of experimental results shows that the FePd nanoparticles have good biocompatibility and photothermal conversion efficiency (36.7%) in NIR-II region. By binding to macrophages, FePd@M has a good tumor targeting ability, and can be quickly and accurately accumulated in tumor site. Subsequently, it can be used for NIR-II photothermal therapy and tumor MRI imaging. The nanoparticles we built overcome the problem of tumor targeting and avoid the rapid removal of ultra-small nanoparticles. The FePd@M delivery system provides new ideas for material construction in the NIR-II region and has a great clinical application potential.

## Conflicts of interest

There are no conflicts of interest to declare.

## Supplementary Material

RA-009-C9RA05649A-s001
