# Supplementary material for: Ultra-small bimetallic iron–palladium (FePd) nanoparticle loaded macrophages for targeted tumor photothermal therapy in NIR-II biowindows and magnetic resonance imaging
Source: RSC Adv. 2019 Oct 17;9(57):33378–87. doi: 10.1039/c9ra05649a (PMC9073278; doi:10.1039/c9ra05649a)
Supplement: RA-009-C9RA05649A-s001 [file RA-009-C9RA05649A-s001.pdf]

Electronic Supplementary Material (ESI) for RSC Advances.
This journal is © The Royal Society of Chemistry 2019

Ultra-small Bimetallic Iron-palladium (FePd) nanoparticles loaded macrophages for targeted tumor photothermal therapy in NIR‑II Biowindows and Magnetic Resonance Imaging

Yang Yang, [§a] Meng Lyu, [§a, c] Jing-Hua Li, [§b] Dao-Ming Zhu, [§a] Yu-Feng Yuan,[*b] and Wei Liu[*a]

aKey Laboratory of Artificial Micro- and Nano-Structures of Ministry of Education, School of Physics and Technology, Wuhan University, Wuhan, Hubei 430072, China.

E-mail: wliu@whu.edu.cn

b Department of Hepatobiliary and Pancreatic Surgery, Zhongnan Hospital of Wuhan University, Wuhan, Hubei 430071, China.

E-mail: yuanyf1971@whu.edu.cn

c Medical Science Research Center, Zhongnan Hospital of Wuhan University, Wuhan, Hubei 430071, China.

§These authors contributed equally to this work.

** To whom correspondence should be addressed.*

Materials

Phosphate buffer solution (PBS) and Dulbecco's modified Eagle's medium (DMEM, Hyclone, high glucose) were obtained from Thermo-Fisher (Waltham, MA, USA). fetal bovine serum (FBS) were purchased from Thermo-Fisher (USA). 1,2-distearoyl-sn-glycero-3-phosphoethanolamine-N-[Cy5(poly-ethylene-glycol)-2000] (ammonium salt) (DSPE-PEG-Cy5) were obtained from Avanti Polar Lipids (USA). All of the aqueous solutions were prepared using deionized (DI) water, which was purified on an experimental water purification system (Direct-Q3, Millipore, USA). The other reagents used in this work were purchased from Sinopharm Chemical Reagent (China) and Aladdin-Reagent (China).

Cell lines processing

Human hepatocarcinoma cell line (Huh-7) was provided by Hospital of Zhongnan Hospital was cultivated in Dulbecco's Modified Eagle's Medium (DMEM), which was supplemented with 10% bovine serum (FBS), and 1% penicillin/streptomycin, at 37°C under a humidified 5% CO_2 atmosphere. The mouse leukocyte cell line (RAW264.7) was provided by College of Life Sciences of Wuhan University was cultured in media supplemented with10% FBS and 1% penicillin-streptomycin at 37°C and 5% CO_2. All the experiments were performed in compliance with the relevant laws and institutional guidelines of China and were

approved by the ethics committee at Wuhan University.

Preparation and Characterization of FePd nanoparticles

FePd nanoparticle were prepared according to the method described previously. In brief, Fe(acac)3(0.12mg) was dissolved in ethanol in a round-bottom flask, after which oleic acid (1.5 ml) and oleyl amine (1.5 ml) was added. Na_2PdCl_4 (10 ml, 3.4 mmol) was slowly added under vigorous stirring and the orange dispersion was stirred for 20 min under nitrogen atmosphere. Next, $NaBH_4$ with certain concentration was dropped in the mixture and reacted at 40℃ for 2 h under vigorous stirring. After removing supernatant, the precipitate was purified by centrifugation and dried in a vacuum oven overnight. The FePd NPs were stored in vacuum oven for further use. The optical absorption spectra of the FePd nanoparticle are acquired on a UV-visible-NIR spectrophotometer. The crystal structures and phase purities of the products were investigated by powder X-ray diffraction (XRD). To figure out the element composition of FePd, energy dispersive X-ray spectroscopy (EDS) was introduced. TEM were acquired at 100 kV was employed to visually the structure of FePd nanoparticles.

In vitro photothermal ability of FePd nanoparticles

To measure photothermal transduction, FePd aqueous solution (0,62.5,125,250,500 ug/ml) were added into the centrifuge tube and then irradiated using a 1064 nm laser source for 3 min. The solution

temperature was measured every 10 s by the Infrared camera. To deduce the photothermal conversion efficiency (η), FePd aqueous solution (500 μg mL^{-1}) was irradiated with NIR laser until the temperature was steady. Then, the laser was turned off and the system temperature was cooled naturally to the ambient temperature with measuring the temperature every 10 s.

We calculated the photothermal conversion efficiency (η) using the reported method.[1] Briefly, the η value was calculated as follows

$$\eta = \frac{hS(T_{max} - T_{surr}) - Q_0}{I(1 - 10^{-A_\lambda})}$$

where h is the heat transfer coefficient, S is the surface area of the container, T_{max} and T_{surr} are the equilibrium temperature and ambient temperature, respectively. Q_0 is the heat associated with the light absorbance of the solvent, A_λ is the absorbance of FePd nanoparticles at 1064 nm, and I is the laser power density. According to the above equation, the η value of FePd nanoparticles was determined to be about 36.7%.

The biocompatibility and cellular uptake of FePd nanoparticles

RAW264.7 cells were cultured on a culture dish with a diameter of 35mm at a density of 1×10^6 cells per dish for 24 h and incubated with different concentrations of FePd nanoparticles (200,300,400,500 ug/mL) and PBS solution for another 24h. Afterward, the cells were contained with 1

mg/mL propidium iodide (PI) and 5 mg/ml fluorescein diacetate (FDA) for 15 min, then washed several times with PBS. The fluorescence microscopy images were acquired on the Olympus IX81 microscope. In the next step, the different concentrations of FePd nanoparticles (200,300,400,500 ug/mL) were lysed with HNO_3 and H_2O_2 mixture solution (HNO_3: H_2O_2 = 3:1) and the Pd concentrations were measured by ICP-AES, at the same time, we added a group 100ug/ml FePd nanoparticles.

RAW264.7 cells were seeded on the wells of a 6-well plate. After incubation for 24 h, adding FePd nanoparticles at different concentrations (0,200,300,400,500 ug/ml) and incubated for another 24 h. After harvesting, an annexin V-FITC and PI apoptosis detection kit were utilized to stain cells (apoptotic cells were labeled by annexin V-FITC and necrotic cells were labeled by PI). And the labeled cells were further determined by Cell Lab Quanta SC flow cytometry quantify the percentage of apoptotic cells and necrotic cells.

Different concentrations of FePd nanoparticles (200,300,400,500 ug/ml) and PBS solution were added and co-cultured with RAW264.7 cells for 0h、24h、36h、48h. At different times, CCK-8 solution was added per instructions, it was incubated for another 2 h. The cytotoxicity was calculated by dividing the optical density (OD) values of treated groups (T) by the OD values of the control (C) (T/C × 100 %).

Animal Experiments

4-week male BALB/c nude mice were managed under protocols approved by Wuhan University. Tumors of mice were prepared with injection of 60 μL of PBS containing 5×10^6 Huh-7 cells into the flanks, and tumor sizes were calculated with the following equation: volume (V) = length × width^2/2.

All animal procedures were performed in accordance with the Guidelines for Care and Use of Laboratory Animals of Wuhan University and Experiments were approved by the Animal Ethics Committee of Wuhan University. All the animals in this study received humane care in compliance with the institution's guidelines for the maintenance and use of laboratory animals in research.

In vivo biodistribution and tumor targeting of FePd@M

Firstly, macrophages and FePd NPs were co-cultured to form FePd@M, and then injected into the mice implanted with human liver cancer cell line Huh-7 tumors. The pure FePd NPs and pure macrophages were injected into the Huh-7 tumor bearing mice as a control group, the FePd@M were injected into the Huh-7 tumor bearing mice as experimental group. In this experiment, materials were labeled with cy5 dye. After 24 hours, Cy5 fluorescence signal were obtained by fluorescence imaging system. At the same time, the tumor-bearing mice of FePd group, macrophages group and FePd@M group were sacrificed.

The tumor and major organs were excised and imaged to obtain the tissue distribution of the Cy5 labeled FePd NPs on an ex/in vivo fluorescence imaging system (Maestro). The Pd centrations were determined by ICP-AES.

In Vitro and in Vivo MRI

FePd nanoparticles were dispersed in saline at different concentrations (16 ug/mL for 0.1mM Fe, 48 ug/mL for 0.3 mM Fe, 96 ug/mL for 0.6 mM Fe, 160 ug/mL for 1.0 mM Fe). Then, the longitudinal and transverse relaxation times were measured on a small animal MRI system. For in vivo MRI imaging. 5×10^6 Huh-7 cells were subcutaneously injected into the flanks of 4-week male BALB/c nude mice. When tumors reached ~400 mm^3, FePd@M (containing 500ug/mL FePd) and FePd (500 ug/mL) were intravenously (i.v.) injected into the mice (n = 5). At 24 h post-injection, the mice were scanned on a 4.7 T Varian small animal MRI system.

In Vivo Photothermal Therapy

5×10^6 Huh-7 cells were subcutaneously injected into the flanks of 4-week male BALB/c nude mice. Analogously, 5×10^6 4T1 cells into the male BALB/c nude mice. Treatments started when tumor size reached ~200 mm^3. 20 mice were randomly divided into 4 groups and were i.v. injected with PBS solution, pure macrophages, FePd nanoparticles (500ug/ml), FePd@M (containing 500 ug/ml FePd). After 24h, the mice bearing

tumors were anesthetized and the entire tumors were exposed to the laser (1064 nm, 1.0 W/cm^2) for 3min. During laser irradiation, an infrared thermal imaging camera was utilized to monitor the temperature changes in the tumor sites. The changes in the tumor volume and body weight post-irradiation were measured every 2 days. And the tumor volume V (mm^3) was calculated according to the formula: Volume = (Tumor length) *(Tumor width) $^2/2$. At 15 days post-injection, the mice were killed, and the tumor tissues were collected and fixed into formalin and were then stained with Ki-67 and terminal deoxynucleotidyl TUNEL and finally examined by using an optical microscope (BX51, Olympus, Japan).

In Vivo Cytotoxicity Evaluation

At 15 days post-injection, the mice were killed, and the tumor tissues were collected and fixed into formalin and were then stained with H&E and finally examined by using an optical microscope (BX51, Olympus, Japan).

Statistical analysis

Statistical evaluations of data were performed using the Student's t test. All results were expressed as mean ± standard error unless otherwise noted. *$P < 0.05$, **$P < 0.01$.

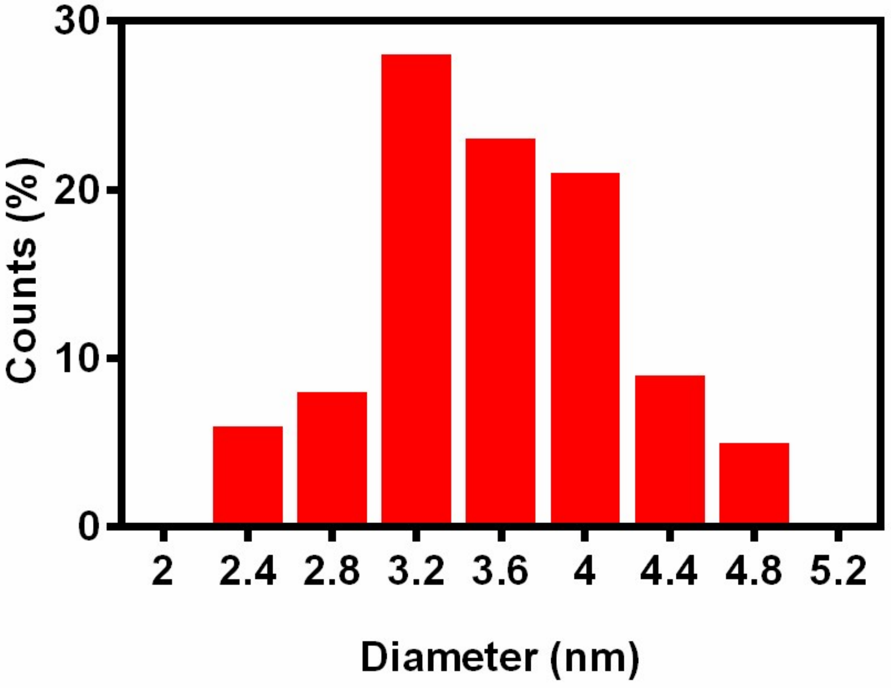

Figure S1. measured diameters of as-synthesized FePd NPs.

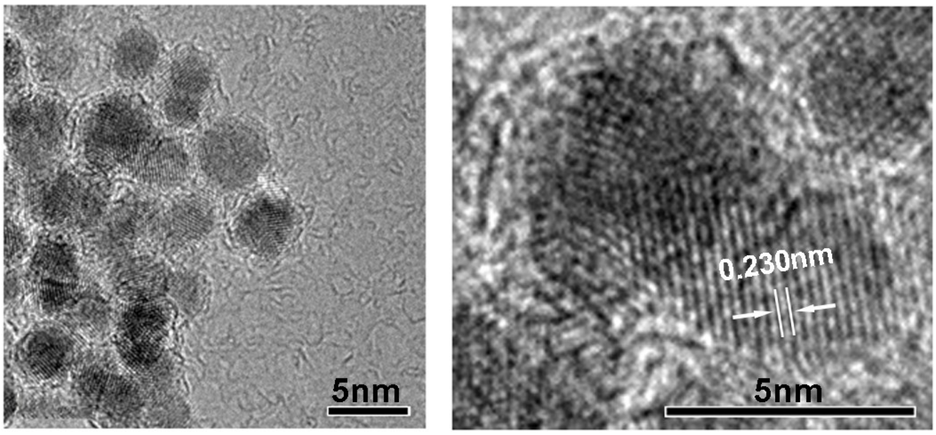

Figure S2. High-resolution TEM image of FePd nanoparticles.

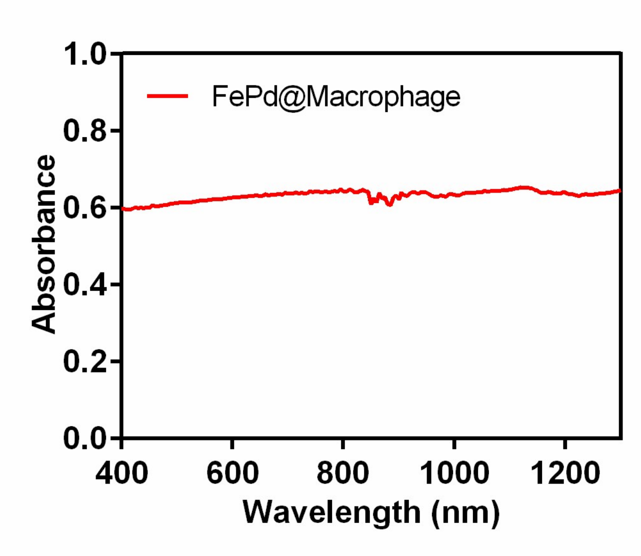

Figure S3. Absorbance spectra of macrophages cultured with FePd nanoparticles for 24h.

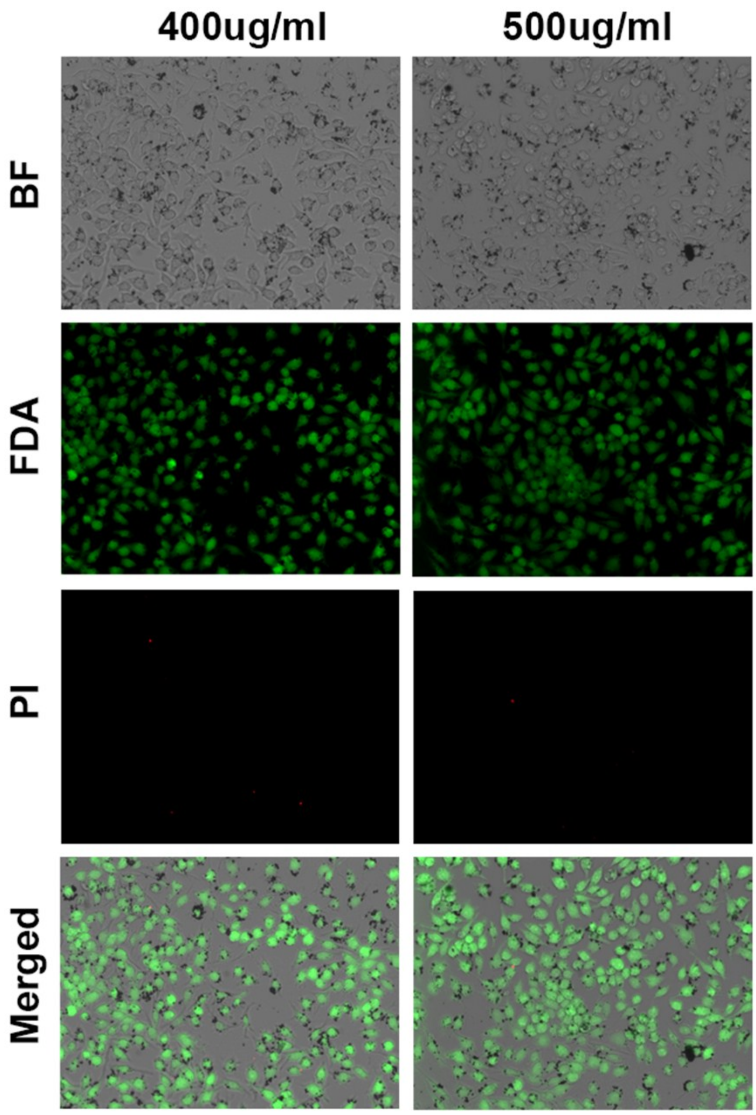

Figure S4. FDA/PI staining (viable cells are stained green with FDA and dead cells are stained red with PI) after the macrophages were incubated with PBS and FePd nanoparticles of different concentration (200, 300, 400, 500 ug/ml) for 48 hours.

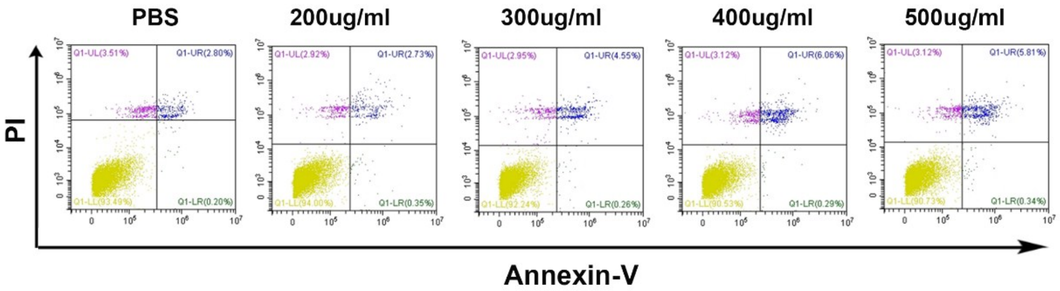

Figure S5. Flow cytometry analysis of macrophages after being incubated with different concentrations of FePd nanoparticles for 24 hours.

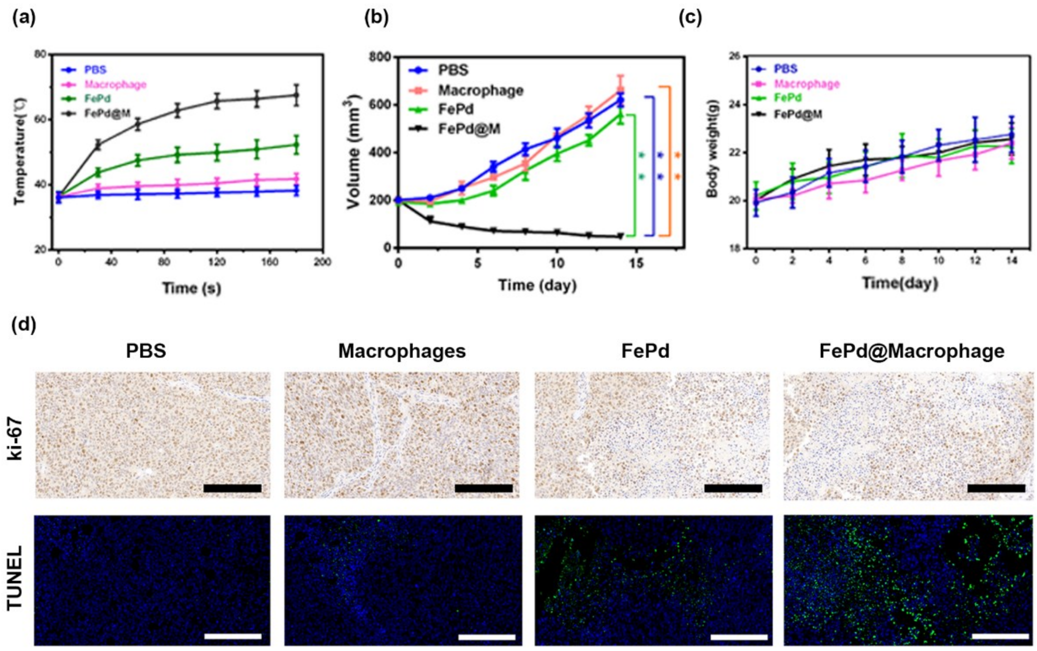

Figure S6. In Vivo Photothermal Therapy and Cytotoxicity. (a) Heating curves of 4T1 tumor-bearing Balb/c mice after injection of PBS, FePd, Macrophages, FePd@Macrophages and exposure to 1064 nm laser irradiation. (b) Tumor volume curves after injection of PBS, FePd, Macrophages, FePd@Macrophages and exposure to 1064 nm laser irradiation. (c) Treatment side effects were assessed by mice body weight in PBS, FePd, Macrophages, FePd@Macrophages group. (d) Representative TUNEL and Ki-67 stained tumor slice images of mice after injection of PBS, FePd, Macrophages, FePd@Macrophages and exposure to 1064 nm laser irradiation. Scale bars = 100 um.

1. D. K. Roper, W. Ahn and M. Hoepfner, *The journal of physical chemistry. C, Nanomaterials and interfaces*, 2007, **111**, 3636-3641.
